# Molecular Structures of the Products of a Diphosphonate Ester Building Block with Lewis Bases

**DOI:** 10.3390/molecules200814435

**Published:** 2015-08-07

**Authors:** Yufeng Li, Fangfang Jian

**Affiliations:** 1 Microscale Science Institute, Weifang University, Weifang 261061, China; E-Mail: liyufeng8111@163.com; 2 New Materials and Function Coordination Chemistry Lab, Qingdao University of Science & Technology, Qingdao 266042, China

**Keywords:** phosphonate ester, synthesis, crystal structure, fluorescence

## Abstract

By treating a suitable Wittig reagent under acid conditions, the phosphonate ester 1,4-bimethylenebenzene phosphonate ethyl ester (H_2_[BBPE], **1**) was obtained. As a building block, compound **1** has been reacted with the Lewis-base *N,N*-dimethylpiperazine, ammonia and NaOH yielded compounds **2**–**4**. The crystal structures show that a 1D chain forming a tubular channel is constructed through hydrogen bonds in **1**; hydrogen bonds form two 1D chains with left-hand and right-hand helixes and form 3D networks in compound **2**; 1D hydrogen-bond chains are further connected together to afford a 3D network architecture in compound **3**; the phosphonate is coordinated by two Na atoms which present different coordination environments in compound **4**. Additionally, the relationships between the structure and fluorescence of the four compounds in the solid state and in different solvents have also been studied at room temperature.

## 1. Introduction

**Scheme 1:**
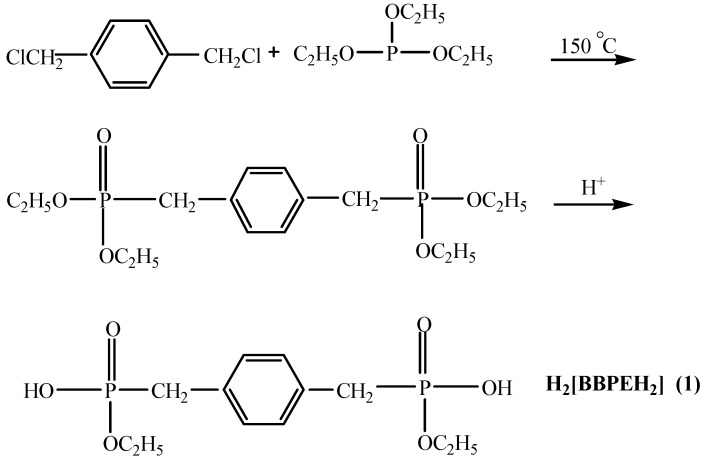
Synthesis of compound **1**.

**Scheme 2:**
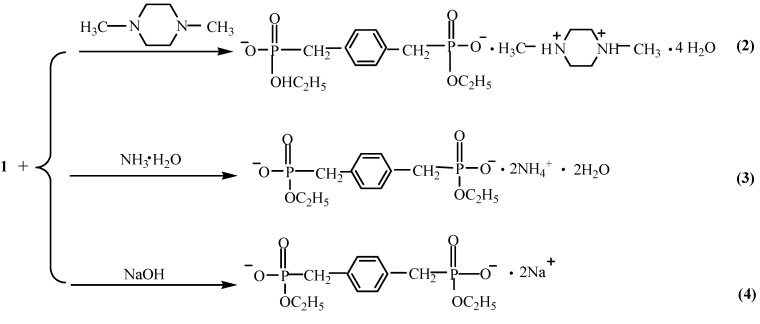
Synthesis of compounds **2**–**4**.

Recently, metal phosphonates have attracted a great deal of research interest as a new class of inorganic-organic materials due to their structural diversity and many potential and practical applications in the fields of nonlinear optics, catalysis, gas storage, ion exchangers and so on [1,2,3,4,5,6,7]. In all these cases, phosphonates play an important role because of their three oxygen donor atoms [8,9,10]. Accordingly, by modifying different organic functional groups, the complexing ability of phosphonates and the corresponding properties of the resulting materials will be changed [11,12,13,14,15,16,17,18,19]. On the other hand, multifunctional phosphonates, such as bisphosphonic acids, aminophosphonic acids and carboxyphosphonic acids, have been proved as good candidates for their potential to form extended hydrogen-bonded assemblies which could be utilized for the preparation of one-, two-, and three-dimensional hydrogen-bonded networks and microporous solids [20,21,22,23,24,25,26,27]. For instance, Yuan *et al.* combined 1-aminoethylidene diphosphonic acid (AEDPH_4_) and 1-aminopropane-1,1,3 triphosphonic acid (APTPH_6_) with 2,2′-bipyridyl-like ligands to build up higher-dimensional supramolecular architectures [3]. Amir *et al.* produced supramolecular isomerism and isomorphism using the structures of 1,4-butanebisphosphonic acid and its organic ammonium salts [2]. In general, single crystals of metal phosphonates are difficult to obtain because of their low solubility, so investigations on the structure of soluble salts of phosphonates are valuable in this field. Sparked by this thought, we designed and synthesized the diphosphonate ester of 1,4-bimethylenebenzene bisphosphonate ethyl ester (H_2_[BBPEH_2_], **1**, where BBPEH_2_ = [O(OC_2_H_5_)(HO)PCH_2_C_6_H_5_CH_2_P(OH) (OC_2_H_5_)O]^2−^} as a bidentate ligand, as shown in Scheme 1. Furthermore, by using a general solution method and room temperature, 1 acted as a building block that easily reacted with organic and inorganic bases, and the formed compounds displayed better solubility in organic solvents or water. We thus obtained compounds **2**–**4** as shown in Scheme 2. As expected, the single crystal structure of two self-assembly acid-base hydrogen-bonded supramolecular architectures and one organic-inorganic hybrid coordination polymer were obtained. Fluorescence spectra of **1**–**4** in the solid-state, in methanol and water solutions have been determined. In this paper, we wish to report the syntheses, crystal structures and fluorescence properties of these four compounds. 

## 2. Results and Discussion

### 2.1. Supramolecular Structure of **1**

**Figure 1:**
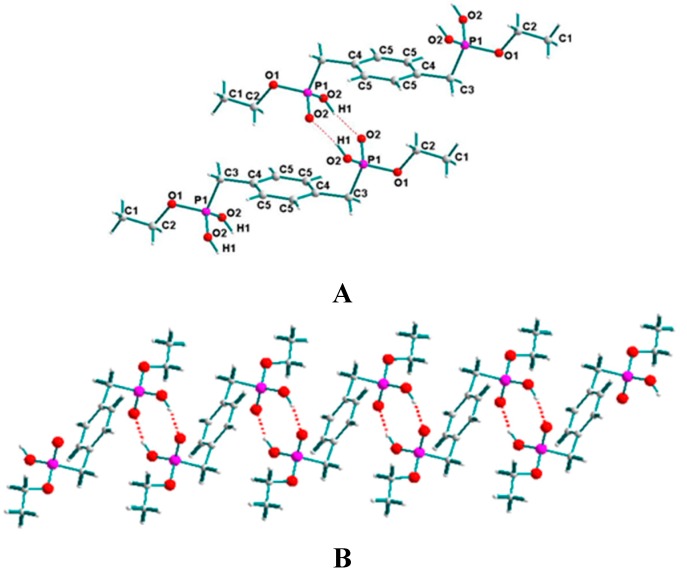
(**A**) Molecular structure of **1** with atomic numbering and (**B**) the molecular arrangement by the hydrogen bonds between adjacent H_2_[BBPEH_2_] molecules.

As depicted in Figure 1A, the unit of **1** contains two independent H_2_[BBPEH_2_] molecules. In each molecule, there exists a 2-fold symmetric axis, which lies in the plane of the phenyl ring and passes through the two middle points of the C5-C5 and C5-C5 bonds. The P-O bond lengths are in the range of 1.5215(16) and 1.581(2) Å, which match those found in the similar structures [13]. In the unit lattice, each pair of adjacent H_2_[BBPEH_2_] molecules are connected each other by two O2-H1···O2 hydrogen bonds and then, a 1D zigzag chain forming a tubular channel is created, just as shown in Figure 1B, with the diameter of the channel being 2.384 Å.

### 2.2. Supramolecular Structure of **2**

**Figure 2:**
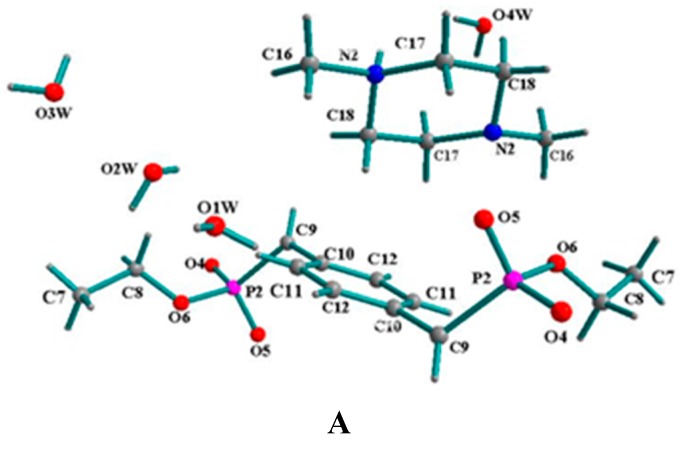

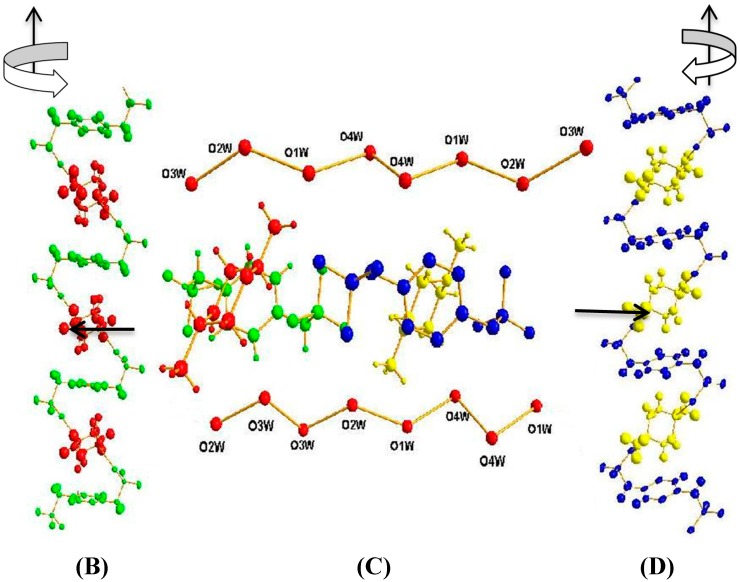
(**A**) Molecular structure of **2** with atomic numbering, (**B**) 1D-R right-handed zigzag chain shown as green-red, (**C**) 3D supramolcular architecture of **2** constructed by water chains and two different symmetry equivalent anion-cation groups and (**D**) 1D-L left-handed zigzag chain shown as blue-yellow.

The molecular structure of **2** with atomic numbering is shown in Figure 2A. A unit cell of compound **2** contains of two [BBPEH_2_]^2−^ anions, two protonated *N,N*-dimethylpiperazine cations and eight lattice water molecules, which are connected together through hydrogen bonds and electrostatic effects. Each H_2_[BBPEH_2_] molecule has lost two protons and transferred them to the *N,N*-dimethylpiperazine nitrogen atoms and form a [BBPEH_2_]^2−^ anion. Both *N,N*-dimethylpiperazine nitrogen atoms are protonated. In compound **2**, there are two types of anion-cation groups with different symmetric equivalence. For one type of anion-cation group, the P1=O1 bond length is 1.444(2) Å, and the P1-O2 and P1-O3 bond lengths are 1.675(2) and 1.689(2) Å, respectively. The [BBPEH_2_]^2−^ anions and protonated *N,N*-dimethylpiperazines are connected each other by N1-H1···O2 hydrogen bonds to form a 1D helical chain, which obeys the right-handed rule along an axis (named 1D-R, shown as green-red in Figure 2B). For the other anion-cation group, the P2=O5 bond length is 1.404 (2) Å, and the P2-O4 and P1-O2 bond lengths are 1.463(2) and 1.693(2) Å, respectively. The [BBPEH_2_]^2−^ anions and protonated *N,N*-dimethylpiperazines are joined each other through N2-H2···O5 hydrogen bonds to afford a 1D helical chain, which obeys the left-handed rule along an axis (named 1D-L, shown as blue-yellow in Figure 2D). Four lattice water molecules play important roles in connecting the 1D chains and further constructing 3D networks (see Figure 2C). The O(2W)-H···O(1) and O(3W)-H···O(2) hydrogen bonds join the two water molecules [H2O2W and H2O3W] with the adjacent 1D-R helix chains, respectively, while at the same time the O(4W)-H···O(5) and O(1W)-H···O(4) hydrogen bonds join the other two water molecules [H2O4W and H2O1W] with the adjacent 1D-L helix chains, respectively. On the other hand, four water molecules are linked one by one through O-H···O hydrogen bonds to form a “water cluster chain”, with the O-O distances in the water cluster ranging between 2.654 and 3.076 Å and the O-H···O bond angles being from 81.49 to 134.63° (see Figure 2C). Finally, the water cluster chains connect all 1D chains together to construct 3D networks in the unit lattice. This 3D networks are further consolidated by some C-H···π interactions (see Table 1)

**Table 1:** Selected C-H···*π* Interactions in Compounds **2**, **3** and **4**.

Compound	C-H···*π*	Symmetry Code	C···*π* Centroid-Centroid (Å)	∠C-H···*π* (°)
**2**	C(14)-H(14B)···Cg(3)	*x*, −1 + *y*, *z*	3.640	149.55
	C(14)-H(14B)···Cg(3)	1− *x*, 1 − *y*, −*z*	3.640	149.55
	C(17)-H(17B)···Cg(4)	*x*, *y*, *z*	3.695	136.52
	C(17)-H(17B)···Cg(4)	− *x*, 1 − *y*, 1 − *z*	3.695	136.52
**3**	C(5)-H(5A)···Cg(1)	− *x*, −½ + *y*, ½ − *z*	4.044	164.92
	C(5)-H(5A)···Cg(1)	*x*, −½ − *y*, ½ + *z*	4.044	164.92
**4**	C(2)-H(2B)···Cg(1)	*x*, *y*, *z*	3.792	145.37
	C(2)-H(2B)···Cg(1)	− *x*, −*y*, −*z*	3.792	145.37

In **2**, Cg(3) denotes phenyl ring containing C(4)~C(6); Cg(4) denotes phenyl ring containing C(10)~C(12); in **3**, Cg(1) denotes phenyl ring containing C(1)~C(3); in **4**, Cg(1) denotes phenyl ring containing C(4)~C(6).

### 2.3. Supramolecular Structure of **3**

**Figure 3:**
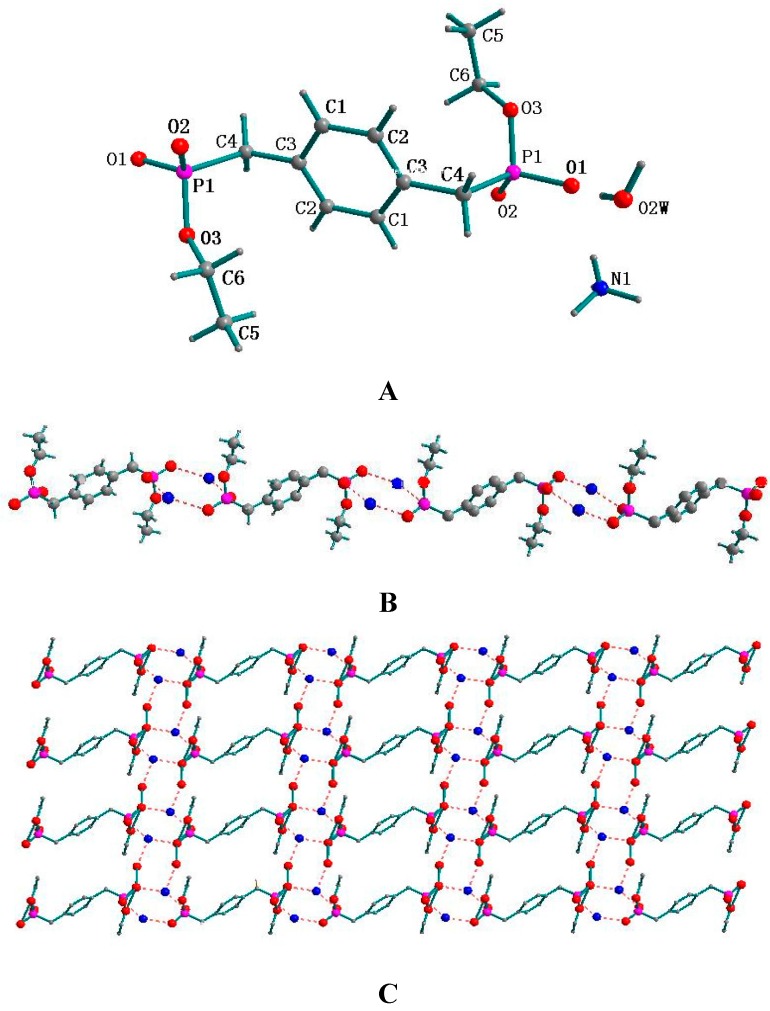

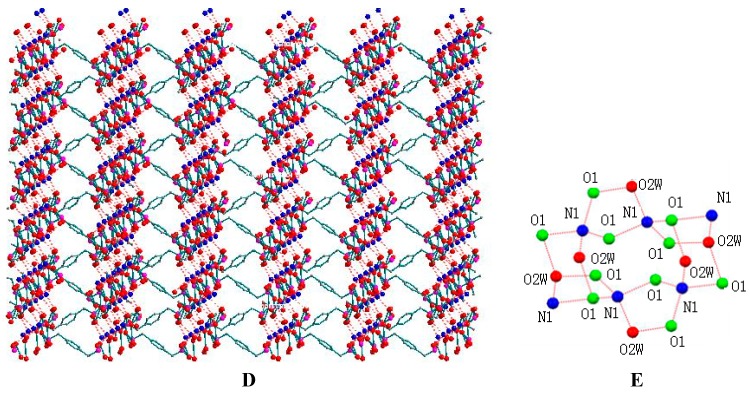
(**A**) Molecular structure of 3 with atomic numbering; (**B**) 1D chain joined through N1-H1···O1 and N1-H6···O2 hydrogen bonds; (**C**) 2D supramolecular network viewing along the c axis in the unit cell; (**D**) view of the 3D architecture and (**E**) stable connection mode among atoms of oxygen and nitrogen.

The molecular structure of **3** with atomic numbering scheme is shown in Figure 3A. A unit cell of **3** contains two [BBPEH_2_]^2−^, four protonated ammonia and four water molecules, which are connected together through hydrogen bonds and electrostatic effects. Just as in **2**, each H_2_[BBPEH_2_] molecule has lost two protons and transferred the protons to two ammonia molecules forming a [BBPEH_2_]^2−^ anion. The nitrogen atoms of ammonia are protonated. The cation-anion ratio is 2:1. As shown in Figure 3B, N(1)-H(1)···O(1) (2.8331 Å) and N(1)-H(6)···O(2) (2.7781 Å) hydrogen bonds firstly join the [BBPEH_2_]^2−^ anions to form a 1D chain, and then, in the unit lattice, all of the 1D chains are linked together forming the 2D array of hydrogen bonds (see Figure 3C) and afford a 3D network architecture through supramolecular interactions (see Figure 3D). For instance, as shown in Figure 3C, two 1D chains are linked together through N-H···O hydrogen bond s. The other 1D chains denoted are joined by the lattice water molecule through O-H···O hydrogen bonds. Further, through N-H···O hydrogen bond, the lattice water molecule and the protonated ammonia molecule are connected, which ultimately make the four 1D chains to build up a 3D network architecture. Most important, by observing the connecting mode among the oxygen and nitrogen atoms in protonated ammonia (see Figure 3E), one can find two six-membered rings and four five-membered rings. 

The cluster aggregation is controlled by a “vertex-connected” and “edge-sharing” association mode [28]. The six-membered rings adopt a “puckered-boat” confirmation. It is evident that these stable five-membered rings and six-membered rings help to strengthen the connection among the above four 1D chains. In the solid state, the C(5)-H(5A)···Cg(1) and C(5)-H(5A)···Cg(1) supramolecular interactions (Table 1) help to stabilize the 3D networks.

### 2.4. Supramolecular Structure of **4**

**Figure 4:**
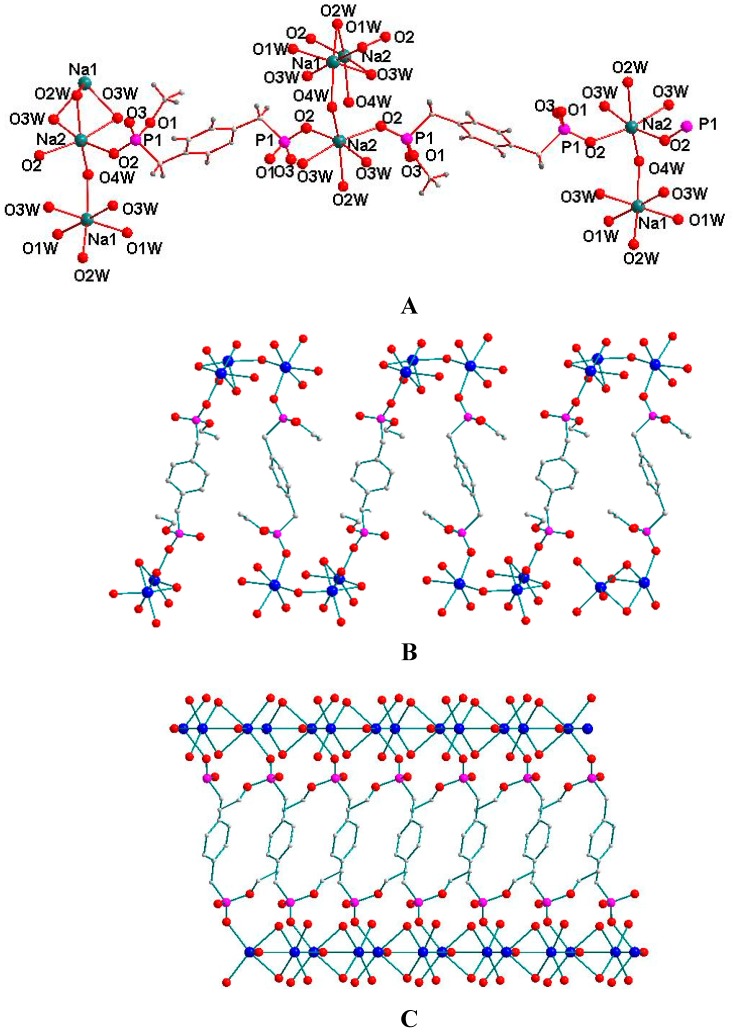
(**A**) Molecule structure of 4 with atomic numbering scheme; (**B**) 2D network in the unit cell and (**C**) 3D network coordination polymer.

The molecular structure of compound **4** with atomic numbering scheme is shown in Figure 4A. The X-ray crystal structural analysis indicates that the repeat unit of compound **4** contains one [BBPEH_2_]^2−^ anion, two sodium cations, and six water molecules. Although the coordination modes can be described as a distorted octahedral geometry, there are two types of Na atoms with different modes. Take Na2 and Na1 cation for instance. Na2 is connected with the Na1 cation through three bridged oxygen atoms (O3W, O3W and O2W) in one direction, whereas in the opposite direction, the Na2 cation is connected with another sodium cation of Na1 only through one bridged oxygen atom of the O4W atom from H2O4W. Each of these Na2 cations is connected with two sodium cations, so for the Na2 cation, its coordination mode can be described by four coordinate oxygen atoms being bridged with two other sodium cations and two coordinated oxygen atoms from two [BBPEH_2_]^2−^ anions. As seen from Figure 4A, for the Na1 cation, its coordination mode is similar to that of Na2, and apart from four bridged oxygen atoms, two coordinated O1W oxygen atoms come from two water molecules but not from two [BBPEH_2_]^2−^ anions as in Na2. As shown in Figure 4A, since each [BBPEH_2_]^2−^ anion is a bidentate ligand, coordinated to two sodium cations Na2 with its two apical O2 oxygen atoms. The Na-O distances are in the 2.413–2.525Å range, which is comparable to that reported for other Na-O bonds [about 2.455 Å] [29]. It is noticeable that, for the same [BBPEH_2_]^2−^ anion, its two coordinated sodium atoms [Na2] adopt the same mode to connect with another two pairs of sodium atoms [Na1], but the two connecting directions are opposite, just as shown in Figure 4B. All the above coordination generates a 3D network coordination polymer. Two types of C-H···π interactions (see Table 1) further consolidate the 3D networks in the lattice.

### 2.5. Fluorescence Properties

**Figure 5:**
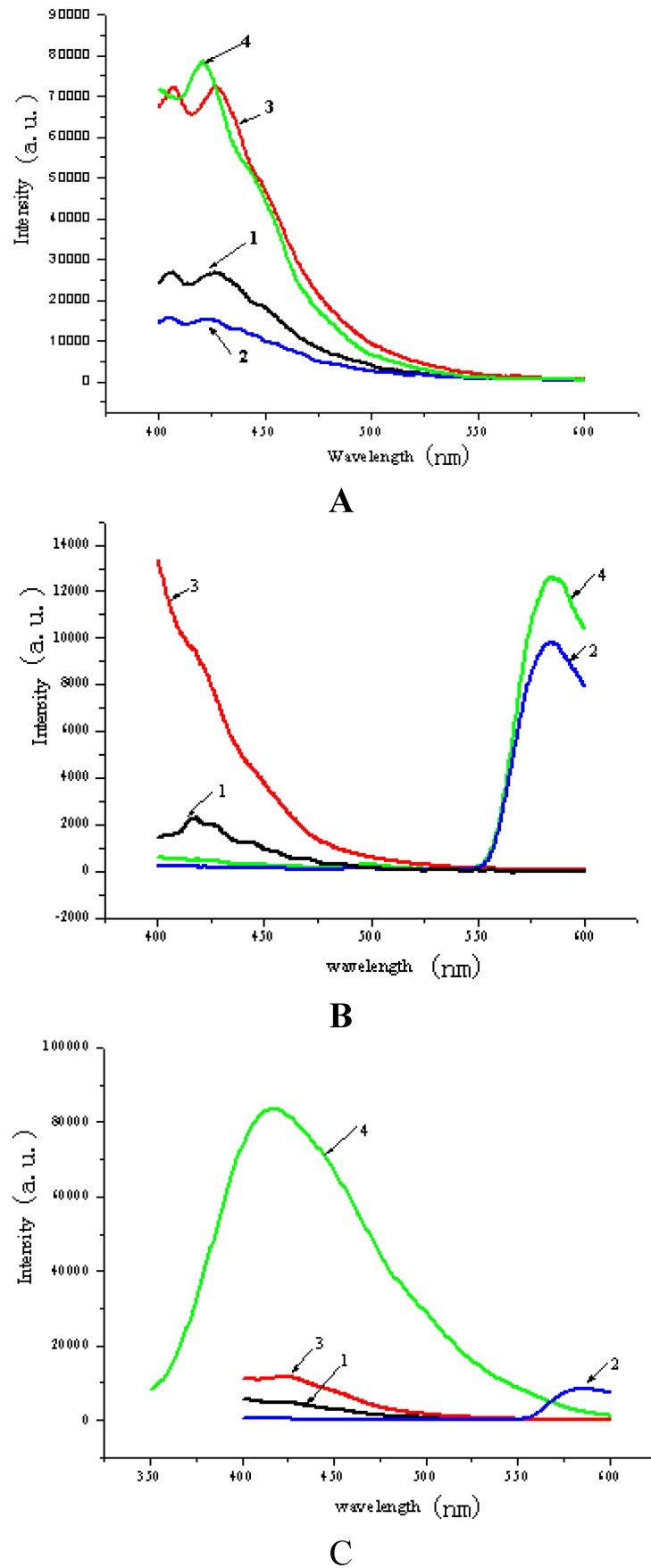
(**A**) The solid-state emission spectra (ex = 356 nm) for **1–4**; (**B**) The emission spectra (ex = 356 nm) for **1–4** in methanol solution and (**C**) The emission spectra (ex = 356 nm) for **1**–**4** in water.

In order to understand the effect of solvents on the crystal structure, the fluorescence spectra for the compounds **1**–**4** in the solid-state, in methanol solution and in water solution at 298 K, upon excitation at 356 nm, have been determined and their fluorescence spectra are shown in Figure 5.

Seen from Figure 5A, in the solid-state, **1** exhibits an emission peak at 426 nm. Under the same experimental conditions, **2**, **3** and **4** exhibit emission peaks at 423, 420 and 428 nm, respectively, which are very similar to that of **1**. These emissions can be tentatively ascribed to an intraligand transition. Based on these similarities, one can deduce that the electron transition modes in **2**, **3** and **4** are very close to that in **1** [30], although the building blocks of [BBPEH_2_]^2−^ have been connected to form a 3D network architecture through hydrogen bonds (in **2** and **3** ) or coordination bonds (in **4**). On the other hand, compound **4** has the strongest fluorescence intensity among **1**–**4**, suggesting that the structure of coordination environment with a Na atom is better for the transfer of electrons than the others. Although **2** and **3** are both supramolecular compounds constructed with the same [BBPEH_2_]^2−^ building block and different organic bases, the fluorescence intensity of **3** is much stronger than that of **2**, which implies that the type of organic base and the type of hydrogen bond in supramolecular compounds have a direct influence on the electron transitions.

From Figure 5B and Figure 5C, for compound **1**, the emission peak in methanol solution is at about 421 nm, which is very near to that in the solid state, indicating the electronic transition mode in methanol solution is the same as that in the solid-state and the supramolecular skeleton of **1** has not been destroyed in the methanol solution. On the other hand, in water solution, no evident emission peak is observed, which suggests that the supramolecular skeleton of **1** in water solution may have collapsed. For **2**, the emission peaks in methanol solution and water solution are both at about 583 nm, which is a 160 nm red shift compared with that in the solid-state, which implies that the supramolecular architecture of **2** has been completely degraded down by the solvents and the electronic transition mode in the solution is different from that in the solid-state. For **3**, in the 400–600 nm band no emission peak observed in methanol solution, while in water solution, there is an emission peak at 423 nm, which differs from that in the solid-state by only 5 nm. For **4**, the emission peak in methanol solution is at about 584 nm, which is a 164 nm red shift compared with that in the solid-state, while in water solution, the emission peak is at 419 nm, which is almost the same with that observed in the solid-state. The phenomena in **3** and **4** reveal that for **3** and **4**, water is better than methanol to maintain the molecular construction, which results in the electronic transition modes in the water solution being the same as those in the solid-state. In methanol solution, the fluorescence spectra of **2** and **4** are red-shifted about 160 nm, indicating that the solid-state skeleton of **2** and **4** has been destroyed, however, their methanol solution still has intense fluorescence. The fluorescence quantum yields of **2** and **4** were measured in methanol at room temperature, using rhodamine B as a standard. Compounds **2** and **4** possessed relative quantum yields of 0.18 and 0.14, respectively, while for **3**, after its supramolecular skeleton is destriyed, no evident emission peak is observed in the 400–600 nm band in the methanol solution of **3**.

Comparing the fluorescence spectra in solution with those in the solid-state, one can find that: (1) the coordination bond is powerful to transfer electrons; (2) solvents have remarkable influences on maintaining the skeletons. Sometimes, the constructs can remain intact in the solvents, such as **3** and **4** in water solution and **1** in methanol solution, and sometimes, solvents will destroy the skeletons, such as **2** in water and methanol solutions.

## 3. Experimental Section

### 3.1. Materials and Methods

All chemicals were of analytical reagent grade and used directly without further purification. Elemental analyses for carbon, hydrogen and nitrogen were performed by a Perkin-Elmer 240 °C Elemental instrument (Waltham, MA, USA). Infrared spectra (4000–400 cm^−1^) were recorded with KBr optics on a Nicolet AVATAR 360 FTIR spectrophotometer (Waltham, MA, USA). The photoluminescence spectra were examined with a HITACHI F-4500 fluorescence spectrophotometer (Tokyo, Japan) equipped with a xenon lamp as the excitation source. Single crystals of this series compounds were obtained in high yield. As a result, all emission data were tested, using single crystals without any impurities as starting materials.

### 3.2. Synthesis of H_2_[BBPEH_2_] (**1**)

The reaction path is shown in Scheme 1. To a toluene solution of 1,4-bis(chloromethyl)benzene (17.5 g, 0.1 mol) was added dropwise triethyl phosphite (33.2 g, 0.2 mol) and the mixture was stirred at 150 °C. Two h later, the reaction mixture was cooled to room temperature. Removal of solvent from the reaction mixture *in vacuo* afforded the corresponding product as a white solid [31] which was further washed with petroleum ether, and finally hydrolyzed in sulfuric acid (5 mol/L) at 40 °C with stirring (yield 86%). The colorless block crystals of 1,4-bismethylenebenzene bisphosphonate ethyl ester (H_2_[BBPEH_2_]) were recrystallized from a mixed solvent of ethanol and distilled water (1:1) one week later. Elemental analysis (%), Calcd. for C_12_H_16_O_6_P_2_: C, 45.28; H, 5.07. Found: C, 45.45; H, 4.89. As the building block, **1** was reacted with the Lewis bases *N,N*-dimethylpiperazine, ammonia and sodium hydroxide. H_2_[BBPEH_2_], has changed from a tetrabasic acid into a binary acid and then, further deprotonated to become the [BBPEH_2_]^2−^ anion. 

### 3.3. Synthesis of [BBPEH2]^2−^·[Protonated N,N-dimethylpiperazine]^2+^·4H_2_O (**2**)

Compound **1** (644 mg, 2.0 mmol) was dissolved in the mixture of water (10.0 mL) and ethanol (10.0 mL) with stirring and then put into a straight glass tube. *N,N*-dimethylpiperazine (0.27 mL, 2.0 mmol) was carefully layered onto it. Colorless block crystals were observed after one week. Elemental analysis (%), Calcd. for C_36_H_80_N_4_O_20_P_4_: C, 42.67; H, 7.96; N, 5.53. Found: C, 42.80; H, 7.85; N, 5.72. 

### 3.4. Synthesis of [BBPEH_2_]^2−^·[NH4]_2_^2+^·2H_2_O (**3**)

The same procedure as for **2** was used. Ammonia (0.160 mL, 4.0 mmol) was used in place of *N,N*-dimethylpiperazine and colorless block crystals were obtained after one week. Elemental analysis (%), Calcd. for C_12_H_30_N_2_O_8_P_2_: C, 36.72; H, 7.71; N, 7.14. Found: C, 36.90; H, 6.89; N, 7.30. 

### 3.5. Synthesis of {[BBPEH_2_]·[Na_2_(H_2_O)_6_]}_∞_ (**4**)

Compound **1** (644 mg, 2.0 mmol) and sodium hydroxide (160 mg, 4.0 mmol) were dissolved in a mixture of water (10.0 mL) and ethanol (10.0 mL) with stirring. Upon slow evaporation of the solvents at room temperature, colorless block single crystals suitable for X-ray analysis were obtained after one week. Elemental analysis (%), Calcd. for C_6_H_9_NaO_6_P: C, 31.17; H, 3.93. Found: C, 32.12; H, 3.73.

### 3.6. Single Crystal X-ray Diffraction Studies

The diffraction data for **1**–**4** were collected on a Enraf-Nonius CAD-4 diffractometer with graphite-monchromated Mo-Kα radiation [λ = 0.71073 Å, T = 293(2) K] by ω-scan mode. The structures were solved by direct methods and refined by least squares on F2 by using the SHELXTL [32] software package. All non-hydrogen atoms were anisotropically refined. The hydrogen atom positions were fixed geometrically at calculated distances and allowed to ride on the parent carbon atoms. The molecular graphics were plotted using SHELXTL. Atomic scattering factors and anomalous dispersion corrections were taken from International Tables for X-ray Crystallography [33]. Further detailed information regarding the crystallographic data and structural analysis for **1**–**4** is listed in Table 2. Table 3 lists the hydrogen bonds in the compounds **1**–**3**. All the bond distances and bond angles of the four compounds are in the normal ranges. Mercury (version 3.0, CCDC, Cambridge, UK) and Dimond 3.0 software (CRYSTAL IMPACT, Bonn, Germany) were used to visualize the structures. Some C-H···π interactions in compounds **2**, **3** and **4** are listed in Table 1.

**Table 2:** Crystal Data and Structure Refinement Summary for Compounds **1**–**4**.

Empirical Formula	C_6_H_8_O_3_P	C_18_H_40_N_2_O_10_P_2_	C_6_ H_15_NO_4_P	C_12_H_30_Na_2_O_12_P_2_
*M*	159.09	506.46	196.16	474.28
crystal size/mm	0.24 × 0.20 × 0.16	0.30 × 0.18 × 0.14	0.22 × 0.20 × 0.18	0.24 × 0.22 × 0.18
*T*/K	293(2)	293(2)	293(2)	293(2)
*λ*/Å	0.71073	0.71073	0.71073	0.71073
crystal system	monoclinic	centric	centric	centric
space group	*C*2	*P*-1	*P*-1	*Pnma*
*a/*Å	13.431(3)	9.3520(19)	13.079(3)	8.2010(16)
*b/*Å	8.8309(18)	10.458(2)	9.1170(18)	28.502(6)
*c*/Å	7.7810(16)	15.736(3)	8.9340(18)	9.7120(19)
*α*/deg	90	81.56(3)	89.88(3)	90
*β*/deg	123.98(3)	82.84(3)	107.82(3)	90
*γ*/deg	90	63.46(3)	90.02(3)	90
*V*/Å^3^	765.3(3)	1358.9(5)	1014.2(4)	2270.1(8)
*Z*	4	2	4	4
*ρ*_calcd_/g.cm^−3^	1.381	1.238	1.285	1.352
*F* (000)	332	544	420	952
*μ/*mm^−1^	0.304	0.208	0.252	0.280
*θ* range/deg	2.94 to 26.98	1.31 to 29.31	1.64 to 26.97	1.43 to 25.00
completeness to *θ*	98.7%	74.9%	97.2%	99.6%
range of *h*, *k*, *l*	−17 ≤ *h* ≤ 14, −11 ≤ *k* ≤ 11, 0 ≤ *l* ≤ 9	0 ≤ *h* ≤ 11, −11 ≤ *k* ≤ 12, −18 ≤ *l* ≤ 18	−15 ≤ *h* ≤ 15, −10 ≤ *k* ≤ 10, 0 ≤ *l* ≤ 10	0 ≤ *h* ≤ 9, −33 ≤ *k* ≤ 0, 0 ≤ *l* ≤ 11
reflections collected/unique	1773/881	6013/5585	4426/2150	2035/2035
*R*_int_	0.0334	0.1221	0.0691	0.0000
data/restraints/parameters	881/1/60	5585/0/297	2150/2/134	2035/0/133
GOF on *F*^2^	1.144	1.027	1.086	1.065
final *R* indices [*I* > 2*σ* (*I*)]	*R*_1_ = 0.0504 *wR*_2_ = 0.1373	*R*_1_ = 0.0574 *wR*_2_ = 0.1688	*R*_1_ = 0.0374 *wR*_2_ = 0.0994	*R*_1_ = 0.0962 *wR*_2_ = 0.2405
*R* indices (all data)	*R*_1_ = 0.0545 *wR*_2_ = 0.1416	*R*_1_ = 0.0954 *wR*_2_ = 0.1946	*R*_1_ = 0.0496 *wR*_2_ = 0.1069	*R*_1_ = 0.1459 *wR*_2_ = 0.2891
peak, hole/e.Å^−3^	0.600 and −0.358	0.584 and −0.610	0.350 and −0.378	1.396 and −1.108

**Table 3:** Selected Hydrogen Bond Lengths (Å) and Angles (°) for Compounds **1**–**3**.

Compound	D-H···A	Symmetry Code	D···A (Å)	∠D-H···A (°)
**1**	O(2)-H(1)···O(2)	− *x*, *y*, −*z*	2.5114	165.95
**2**	N(1)-H(1)···O(2)	*x*, −1 + *y*, *z*	2.6265	175.86
	O(1w)-H(11W)···O(4W)	−1 + *x*, *y*, *z*	3.0756	126.99
	O(2W)-H(12W)···O(1)	−1 + *x*, *y*, *z*	2.8627	155.48
	O(3W)-H(13W)···O(2)	2 − *x*, 1 − *y*, −*z*	3.0145	164.35
	O(4W)-H(14W)···O(5)	1 − *x*, 1 − *y*, 1 − *z*	2.9162	172.15
	O(1W)-H(21W)···O(4)	*x*, −1 + *y*, *z*	2.8216	164.14
**3**	N (1)-H(1)···O(1)	*x*, ½ − *y*, ½ + z	2.8338	168.93
	O(2W)-H(3)···O(2)	*x*, ½ − *y*, ½ + *z*	2.7913	171.10
	N(1)-H(5)···O(2W)	1 − *x*, −1/2 + *y*, ½ − *z*	2.8304	162.13
	N(1)-H(6)···O(2)	1 − *x*, ½ + *y*, ½ − *z*	2.7767	169.00

## 4. Conclusions

By using **1** as the building block, hydrogen-bonded supramolecules and coordinated polymers have been obtained by reaction with Lewis bases. X-ray single crystal diffraction shows that the differences in the structures of the in/organic bases lead to organophosphonic ester supramolecular and coordination polymer structures ranging from 1D helix chains to 3D networks. Solid state fluorescence spectra show that the fluorescence intensities of **1**–**4** are very different from each other, which indicate that the different types of base in the compounds have direct effects on the fluorescence intensity. Fluorescence spectra in solutions indicate that solvents have remarkable influencs on maintaining the integrity of the molecular skeletons. In a larger sense, compound **1** with multiple phosphonate groups can be used to construct higher-dimensional and stable supramolecular architectures extending other alkalis because of its good solubility. In additional, they can be used as models to design new metal phosphonates and explore the variety of structural possibilities in forming organic-inorganic hybrid materials and metal-organic frameworks. Work is currently underway to synthesize metal organophosphonic esters and study the correlation factors that affect the structures and properties.

## Supplementary Materials

The crystallographic file in cif format for **1**–**4** compounds has been deposited with CCDC number 710324–710327. These data can be obtained free of charge via http://www.ccdc.cam.ac.uk/conts/retrieving.html. Supplementary materials can be accessed at: http://www.mdpi.com/1420-3049/20/08/14435/s1.
