# Supplementary material for: Molecular Structures of the Products of a Diphosphonate Ester Building Block with Lewis Bases
Source: Molecules. 2015 Aug 7;20(8):14435–50. doi: 10.3390/molecules200814435 (PMC6332391; doi:10.3390/molecules200814435)

checkCIF/PLATON report

You have not supplied any structure factors. As a result the full set of tests cannot be run.

THIS REPORT IS FOR GUIDANCE ONLY. IF USED AS PART OF A REVIEW PROCEDURE FOR PUBLICATION, IT SHOULD NOT REPLACE THE EXPERTISE OF AN EXPERIENCED CRYSTALLOGRAPHIC REFEREE.

No syntax errors found.   CIF dictionary   Interpreting this report

Datablock: qj0791

```
Bond precision:  C-C = 0.0054 A          Wavelength=0.71073

Cell:           a=9.3520(19)      b=10.458(2)      c=15.736(3)
                alpha=81.56(3)    beta=82.84(3)    gamma=63.46(3)
Temperature:    293 K

                Calculated                 Reported
Volume          1358.9(6)                  1358.9(5)
Space group     P -1                       P-1
Hall group      -P 1                       ?
                C12 H18 O6 P2, C12 H16 O6
Moiety formula  P2, C6 H14 N2, C6 H16 N2,  ?
                8(H2 O)
Sum formula     C36 H80 N4 O20 P4          C36 H80 N4 O20 P4
Mr              1012.92                    1012.92
Dx,g cm-3       1.238                      1.238
Z               1                          1
Mu (mm-1)       0.208                      0.208
F000            544.0                      544.0
F000'           544.70
h,k,lmax        11,12,19                   11,12,18
Nref            5070                       4902
Tmin,Tmax       0.970,0.983
Tmin'           0.969

Correction method= Not given

Data completeness= 0.967          Theta(max)= 25.500

R(reflections)= 0.0570( 3313)     wR2(reflections)= 0.1925( 4902)

S = 1.053                  Npar= 297
```

The following ALERTS were generated. Each ALERT has the format
test-name_ALERT_alert-type_alert-level.
Click on the hyperlinks for more details of the test.

Alert level B

```
PLAT415_ALERT_2_B Short Inter D-H..H-X       H2W3    ..   H7A      ..        2.08 Ang.
PLAT420_ALERT_2_B D-H Without Acceptor       O2W     -    H2W2    ...      Please Check
PLAT420_ALERT_2_B D-H Without Acceptor       O3W     -    H2W3    ...      Please Check
PLAT420_ALERT_2_B D-H Without Acceptor       O4W     -    H2W4    ...      Please Check
```

Alert level C

```
RINTA01_ALERT_3_C  The value of Rint is greater than 0.12
            Rint given   0.122
PLAT029_ALERT_3_C _diffrn_measured_fraction_theta_full Low .......      0.967 Note
PLAT052_ALERT_1_C Info on Absorption Correction Method Not Given .     Please Do !
PLAT220_ALERT_2_C Large Non-Solvent  C     Ueq(max)/Ueq(min) Range        3.1 Ratio
PLAT250_ALERT_2_C Large U3/U1 Ratio for Average U(i,j) Tensor ....        2.5 Note
PLAT340_ALERT_3_C Low Bond Precision on  C-C Bonds ...............     0.0054 Ang.
PLAT352_ALERT_3_C Short   N-H (X0.87,N1.01A) N2      -    H2      ...       0.74 Ang.
PLAT363_ALERT_2_C Long   C(sp3)-C(sp2) Bond  C14     -    C15_c   ...       1.64 Ang.
```

Alert level G

```
PLAT005_ALERT_5_G No _iucr_refine_instructions_details  in the CIF     Please Do !
PLAT007_ALERT_5_G Number of Unrefined Donor-H Atoms .............          8 Report
PLAT072_ALERT_2_G SHELXL First  Parameter in WGHT Unusually Large.       0.12 Report
PLAT154_ALERT_1_G The su's on the Cell Angles are Equal ..........    0.03000 Degree
PLAT199_ALERT_1_G Reported _cell_measurement_temperature ..... (K)        293 Check
PLAT200_ALERT_1_G Reported   _diffrn_ambient_temperature ..... (K)        293 Check
PLAT333_ALERT_2_G Check Large Av C6-Ring C-C Dist. C4     -C6_a           1.44 Ang.
PLAT335_ALERT_2_G Check Large C6 Ring    C-C Range C4     -C6_a           0.15 Ang.
PLAT432_ALERT_2_G Short Inter X...Y Contact  O6      ..  C16      ..      3.00 Ang.
PLAT720_ALERT_4_G Number of Unusual/Non-Standard Labels ..........          8 Note
PLAT764_ALERT_4_G Overcomplete CIF Bond List Detected (Rep/Expd) .       1.14 Ratio
PLAT793_ALERT_4_G The Model has Chirality at N1      .............          R Verify
PLAT899_ALERT_4_G SHELXL97   is Deprecated and Succeeded by SHELXL       2014 Note
```

---

0 **ALERT level A** = Most likely a serious problem - resolve or explain
4 **ALERT level B** = A potentially serious problem, consider carefully
8 **ALERT level C** = Check. Ensure it is not caused by an omission or oversight
13 **ALERT level G** = General information/check it is not something unexpected

4 ALERT type 1 CIF construction/syntax error, inconsistent or missing data
11 ALERT type 2 Indicator that the structure model may be wrong or deficient
4 ALERT type 3 Indicator that the structure quality may be low
4 ALERT type 4 Improvement, methodology, query or suggestion
2 ALERT type 5 Informative message, check

It is advisable to attempt to resolve as many as possible of the alerts in all categories. Often the minor alerts point to easily fixed oversights, errors and omissions in your CIF or refinement strategy, so attention to these fine details can be worthwhile. In order to resolve some of the more serious problems it may be necessary to carry out additional measurements or structure refinements. However, the purpose of your study may justify the reported deviations and the more serious of these should normally be commented upon in the discussion or experimental section of a paper or in the "special_details" fields of the CIF. checkCIF was carefully designed to identify outliers and unusual parameters, but every test has its limitations and alerts that are not important in a particular case may appear. Conversely, the absence of alerts does not guarantee there are no aspects of the results needing attention. It is up to the individual to critically assess their own results and, if necessary, seek expert advice.

Publication of your CIF in IUCr journals

A basic structural check has been run on your CIF. These basic checks will be run on all CIFs submitted for publication in IUCr journals (*Acta Crystallographica*, *Journal of Applied Crystallography*, *Journal of Synchrotron Radiation*); however, if you intend to submit to *Acta Crystallographica Section C* or *E*, you should make sure that full publication checks are run on the final version of your CIF prior to submission.

Publication of your CIF in other journals

Please refer to the *Notes for Authors* of the relevant journal for any special instructions relating to CIF submission.

PLATON version of 20/08/2014; check.def file version of 18/08/2014

Datablock qj0791 - ellipsoid plot

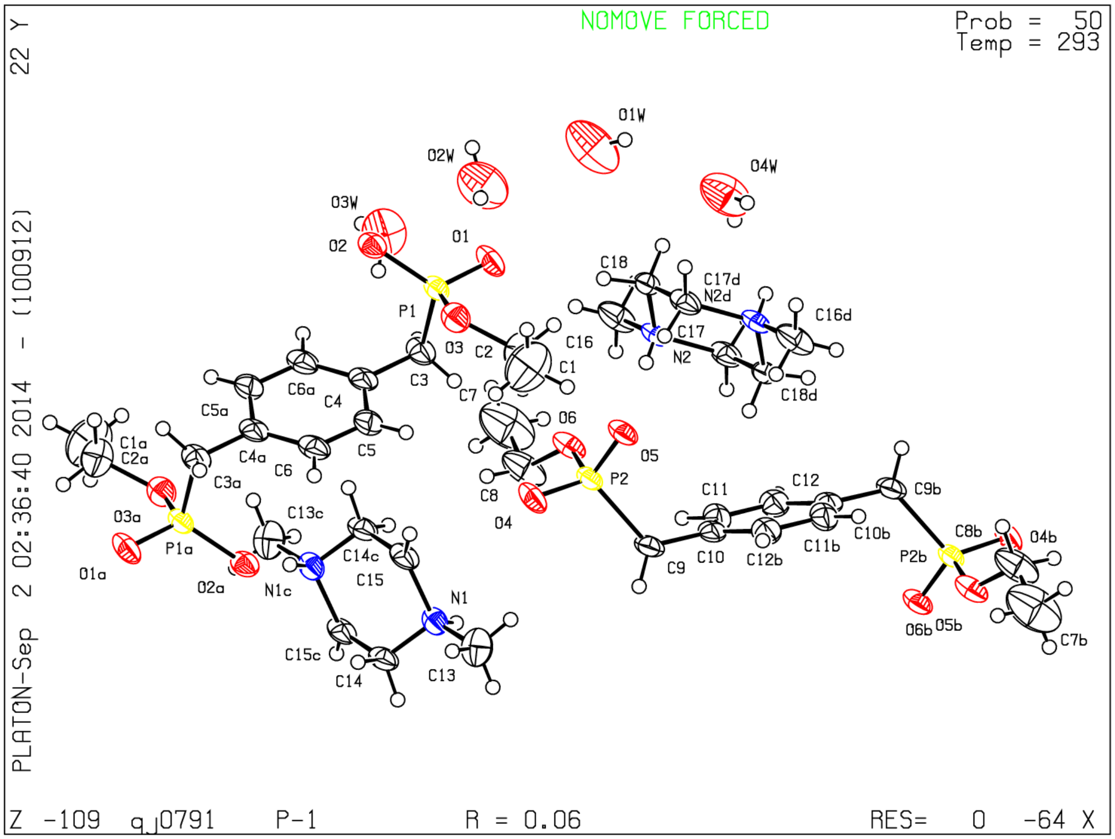

Supplement: Supplementary file 1 [file molecules-20-14435-s001.zip › cif/2-check.pdf]
