# Supplementary material for: Molecular Structures of the Products of a Diphosphonate Ester Building Block with Lewis Bases
Source: Molecules. 2015 Aug 7;20(8):14435–50. doi: 10.3390/molecules200814435 (PMC6332391; doi:10.3390/molecules200814435)

checkCIF/PLATON report

You have not supplied any structure factors. As a result the full set of tests cannot be run.

THIS REPORT IS FOR GUIDANCE ONLY. IF USED AS PART OF A REVIEW PROCEDURE FOR PUBLICATION, IT SHOULD NOT REPLACE THE EXPERTISE OF AN EXPERIENCED CRYSTALLOGRAPHIC REFEREE.

No syntax errors found.   CIF dictionary   Interpreting this report

Datablock: qj0745

Bond precision: C-C = 0.0030 A   Wavelength=0.71073

Cell:	a=13.079(3)	b=9.1170(18)	c=8.9340(18)
	alpha=90	beta=107.82(3)	gamma=90
Temperature:	293 K		

	Calculated	Reported
Volume	1014.2(4)	1014.2(4)
Space group	P 21/c	P21/c
Hall group	-P 2ybc	?
Moiety formula	C12 H18 O6 P2, 2(H2 O), 2(H4 N)	?
Sum formula	C12 H30 N2 O8 P2	C12 H30 N2 O8 P2
Mr	392.32	392.32
Dx,g cm-3	1.285	1.285
Z	2	2
Mu (mm-1)	0.252	0.252
F000	420.0	420.0
F000’	420.63	
h,k,lmax	16,11,11	15,10,10
Nref	2216	2150
Tmin,Tmax	0.946,0.956	
Tmin’	0.946	

Correction method= Not given

Data completeness= 0.970   Theta(max)= 26.990

R(reflections)= 0.0374(1741)   wR2(reflections)= 0.1068(2150)

S = 1.086   Npar= 134

The following ALERTS were generated. Each ALERT has the format
test-name_ALERT_alert-type_alert-level.
Click on the hyperlinks for more details of the test.

Alert level C

```
PLAT029_ALERT_3_C _diffrn_measured_fraction_theta_full Low .......      0.970 Note
PLAT147_ALERT_1_C su on Symmetry Constrained Cell Angle(s) .......     Please Check
```

Alert level G

```
PLAT002_ALERT_2_G Number of Distance or Angle Restraints on AtSite          3 Note
PLAT005_ALERT_5_G No _iucr_refine_instructions_details  in the CIF     Please Do !
PLAT199_ALERT_1_G Reported _cell_measurement_temperature ..... (K)        293 Check
PLAT200_ALERT_1_G Reported   _diffrn_ambient_temperature ..... (K)        293 Check
PLAT860_ALERT_3_G Number of Least-Squares Restraints ............           2 Note
PLAT899_ALERT_4_G SHELXL97   is Deprecated and Succeeded by SHELXL       2014 Note
```

```
   0 ALERT level A = Most likely a serious problem - resolve or explain
   0 ALERT level B = A potentially serious problem, consider carefully
   2 ALERT level C = Check. Ensure it is not caused by an omission or oversight
   6 ALERT level G = General information/check it is not something unexpected

   3 ALERT type 1 CIF construction/syntax error, inconsistent or missing data
   1 ALERT type 2 Indicator that the structure model may be wrong or deficient
   2 ALERT type 3 Indicator that the structure quality may be low
   1 ALERT type 4 Improvement, methodology, query or suggestion
   1 ALERT type 5 Informative message, check
```

It is advisable to attempt to resolve as many as possible of the alerts in all categories. Often the minor alerts point to easily fixed oversights, errors and omissions in your CIF or refinement strategy, so attention to these fine details can be worthwhile. In order to resolve some of the more serious problems it may be necessary to carry out additional measurements or structure refinements. However, the purpose of your study may justify the reported deviations and the more serious of these should normally be commented upon in the discussion or experimental section of a paper or in the "special_details" fields of the CIF. checkCIF was carefully designed to identify outliers and unusual parameters, but every test has its limitations and alerts that are not important in a particular case may appear. Conversely, the absence of alerts does not guarantee there are no aspects of the results needing attention. It is up to the individual to critically assess their own results and, if necessary, seek expert advice.

Publication of your CIF in IUCr journals

A basic structural check has been run on your CIF. These basic checks will be run on all CIFs submitted for publication in IUCr journals (*Acta Crystallographica*, *Journal of Applied Crystallography*, *Journal of Synchrotron Radiation*); however, if you intend to submit to *Acta Crystallographica Section C* or *E*, you should make sure that full publication checks are run on the final version of your CIF prior to submission.

Publication of your CIF in other journals

Please refer to the *Notes for Authors* of the relevant journal for any special instructions relating to CIF submission.

PLATON version of 20/08/2014; check.def file version of 18/08/2014

Datablock qj0745 - ellipsoid plot

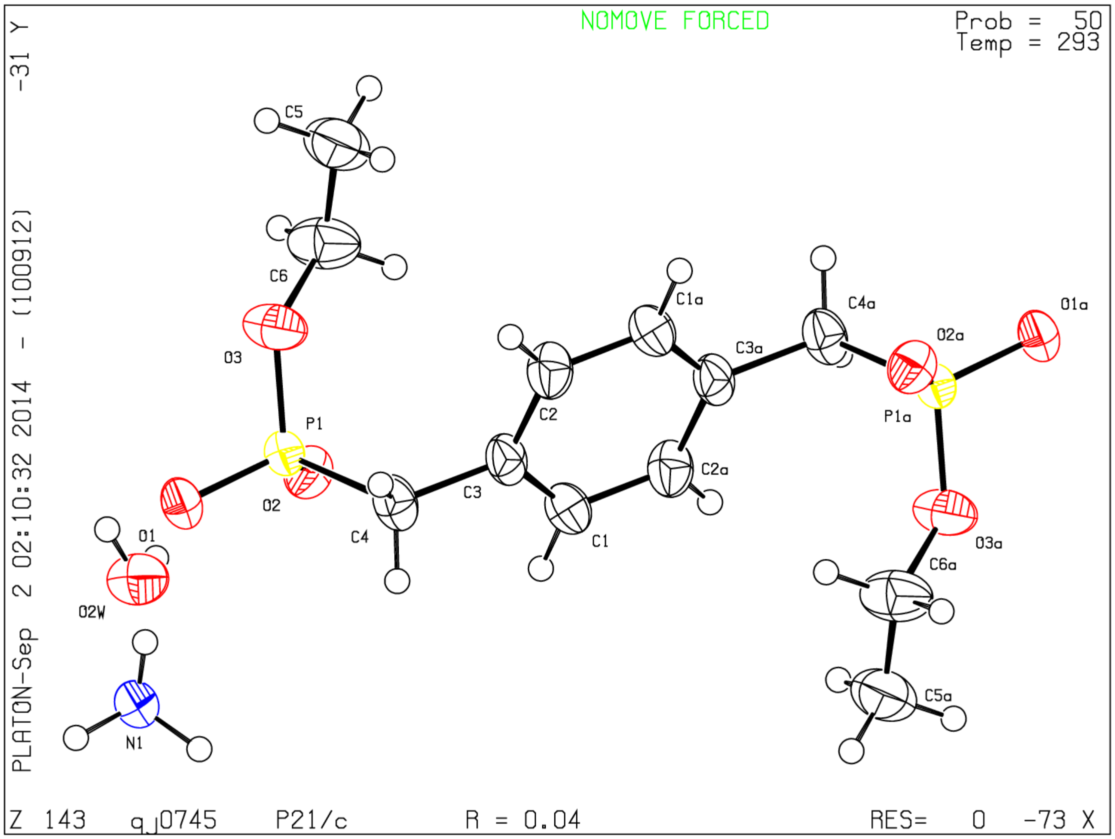

Supplement: Supplementary file 1 [file molecules-20-14435-s001.zip › cif/3-check.pdf]
