# Supplementary material for: Molecular Structures of the Products of a Diphosphonate Ester Building Block with Lewis Bases
Source: Molecules. 2015 Aug 7;20(8):14435–50. doi: 10.3390/molecules200814435 (PMC6332391; doi:10.3390/molecules200814435)

checkCIF/PLATON report

You have not supplied any structure factors. As a result the full set of tests cannot be run.

THIS REPORT IS FOR GUIDANCE ONLY. IF USED AS PART OF A REVIEW PROCEDURE FOR PUBLICATION, IT SHOULD NOT REPLACE THE EXPERTISE OF AN EXPERIENCED CRYSTALLOGRAPHIC REFEREE.

No syntax errors found. CIF dictionary Interpreting this report

Datablock: qj0737

```
Bond precision:  C-C = 0.0092 A          Wavelength=0.71073

Cell:             a=8.2010(16)      b=28.502(6)     c=9.7120(19)
                  alpha=90          beta=90         gamma=90
Temperature:      293 K

                Calculated                 Reported
Volume          2270.1(8)                  2270.1(8)
Space group     P n m a                    Pnma
Hall group      -P 2ac 2n                  ?
Moiety formula  C6 H9 Na O6 P              C6 H9 Na O6 P
Sum formula     C6 H9 Na O6 P              C6 H9 Na O6 P
Mr              231.09                     231.09
Dx,g cm-3       1.352                      1.352
Z               8                          8
Mu (mm-1)       0.280                      0.280
F000            952.0                      952.0
F000'           953.64
h,k,lmax        9,33,11                    9,33,11
Nref            2043                       2035
Tmin,Tmax       0.935,0.951
Tmin'           0.932

Correction method= Not given

Data completeness= 0.996          Theta(max)= 25.000

R(reflections)= 0.0962( 1239)     wR2(reflections)= 0.2891( 2035)

S = 1.065                   Npar= 133
```

The following ALERTS were generated. Each ALERT has the format
test-name_ALERT_alert-type_alert-level.
Click on the hyperlinks for more details of the test.

Alert level B

```
PLAT430_ALERT_2_B Short Inter D...A Contact  O1W    ..  O2W     ..        2.80 Ang.
PLAT430_ALERT_2_B Short Inter D...A Contact  O1W    ..  O3      ..        2.81 Ang.
PLAT430_ALERT_2_B Short Inter D...A Contact  O1W    ..  O3      ..        2.84 Ang.
PLAT430_ALERT_2_B Short Inter D...A Contact  O2     ..  O4W     ..        2.83 Ang.
```

Alert level C

```
DIFMX01_ALERT_2_C  The maximum difference density is > 0.1*ZMAX*0.75
            _refine_diff_density_max given =      1.396
            Test value =      1.125
DIFMX02_ALERT_1_C  The maximum difference density is > 0.1*ZMAX*0.75
            The relevant atom site should be identified.
RFACR01_ALERT_3_C  The value of the weighted R factor is > 0.25
            Weighted R factor given   0.289
PLAT084_ALERT_3_C High wR2 Value (i.e. > 0.25) ...................       0.29 Report
PLAT097_ALERT_2_C Large Reported Max.  (Positive) Residual Density       1.40 eA-3
PLAT250_ALERT_2_C Large U3/U1 Ratio for Average U(i,j) Tensor ....        2.6 Note
PLAT340_ALERT_3_C Low Bond Precision on  C-C Bonds ...............     0.0092 Ang.
```

Alert level G

```
PLAT004_ALERT_5_G Polymeric Structure Found with Dimension .......          3 Info
PLAT005_ALERT_5_G No _iucr_refine_instructions_details  in the CIF     Please Do !
PLAT072_ALERT_2_G SHELXL First  Parameter in WGHT Unusually Large.       0.20 Report
PLAT199_ALERT_1_G Reported _cell_measurement_temperature ..... (K)        293 Check
PLAT200_ALERT_1_G Reported   _diffrn_ambient_temperature ..... (K)        293 Check
PLAT764_ALERT_4_G Overcomplete CIF Bond List Detected (Rep/Expd) .       1.23 Ratio
PLAT774_ALERT_1_G Suspect X-Y Bond in CIF:    NA1    --  NA2     ..       4.21 Ang.
PLAT774_ALERT_1_G Suspect X-Y Bond in CIF:    NA2    --  NA1     ..       4.21 Ang.
PLAT793_ALERT_4_G The Model has Chirality at P1      .............          R Verify
PLAT899_ALERT_4_G SHELXL97   is Deprecated and Succeeded by SHELXL       2014 Note
```

```
   0 ALERT level A = Most likely a serious problem - resolve or explain
   4 ALERT level B = A potentially serious problem, consider carefully
   7 ALERT level C = Check. Ensure it is not caused by an omission or oversight
  10 ALERT level G = General information/check it is not something unexpected

   5 ALERT type 1 CIF construction/syntax error, inconsistent or missing data
   8 ALERT type 2 Indicator that the structure model may be wrong or deficient
   3 ALERT type 3 Indicator that the structure quality may be low
   3 ALERT type 4 Improvement, methodology, query or suggestion
   2 ALERT type 5 Informative message, check
```

It is advisable to attempt to resolve as many as possible of the alerts in all categories. Often the minor alerts point to easily fixed oversights, errors and omissions in your CIF or refinement strategy, so attention to these fine details can be worthwhile. In order to resolve some of the more serious problems it may be necessary to carry out additional measurements or structure refinements. However, the purpose of your study may justify the reported deviations and the more serious of these should normally be commented upon in the discussion or experimental section of a paper or in the "special_details" fields of the CIF. checkCIF was carefully designed to identify outliers and unusual parameters, but every test has its limitations and alerts that are not important in a particular case may appear. Conversely, the absence of alerts does not guarantee there are no aspects of the results needing attention. It is up to the individual to critically assess their own results and, if necessary, seek expert advice.

Publication of your CIF in IUCr journals

A basic structural check has been run on your CIF. These basic checks will be run on all CIFs submitted for publication in IUCr journals (*Acta Crystallographica*, *Journal of Applied Crystallography*, *Journal of Synchrotron Radiation*); however, if you intend to submit to *Acta Crystallographica Section C* or *E*, you should make sure that full publication checks are run on the final version of your CIF prior to submission.

Publication of your CIF in other journals

Please refer to the *Notes for Authors* of the relevant journal for any special instructions relating to CIF submission.

PLATON version of 20/08/2014; check.def file version of 18/08/2014

Datablock qj0737 - ellipsoid plot

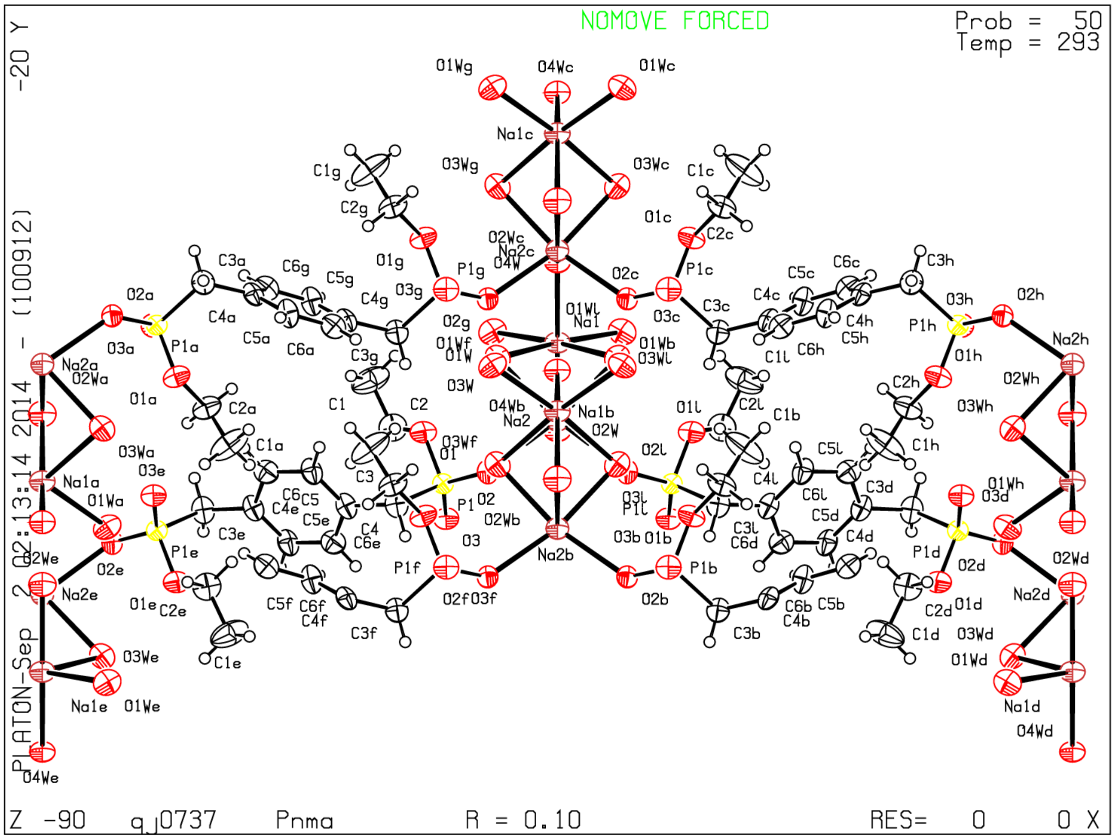

Supplement: Supplementary file 1 [file molecules-20-14435-s001.zip › cif/4-check.pdf]
